# Does Inflammation Contribute to Cancer Incidence and Mortality during Aging? A Conceptual Review

**DOI:** 10.3390/cancers14071622

**Published:** 2022-03-23

**Authors:** Florent Guerville, Isabelle Bourdel-Marchasson, Julie Déchanet-Merville, Isabelle Pellegrin, Pierre Soubeyran, Victor Appay, Maël Lemoine

**Affiliations:** 1 ImmunoConcEpT, CNRS UMR5164, INSERM ERL1303, Université de Bordeaux, F-33076 Bordeaux, France; julie.dechanet-merville@u-bordeaux.fr (J.D.-M.); isabelle.pellegrin@chu-bordeaux.fr (I.P.); vappay@immuconcept.org (V.A.); mlemoine@immuconcept.org (M.L.); 2 Clinical Gerontology Department, Bordeaux University Hospital, F-33000 Bordeaux, France; isabelle.bourdel-marchasson@chu-bordeaux.fr; 3 CRMSB, CNRS UMR 5536, Université de Bordeaux, F-33000 Bordeaux, France; 4 Laboratory of Immunology and Immunogenetics, Bordeaux University Hospital, F-33000 Bordeaux, France; 5 Department of Medical Oncology, Institut Bergonie, F-33076 Bordeaux, France; p.soubeyran@bordeaux.unicancer.fr

**Keywords:** aging, older persons, cancer, inflammation, cell senescence, biomarkers

## Abstract

**Simple Summary:**

Aging is associated with chronic low-grade inflammation, cancer incidence and mortality. As inflammation contributes to cancer initiation and progression, one could hypothesize that age-associated chronic low-grade inflammation contributes to the increase in cancer incidence and/or mortality observed during aging. Here, we review the epidemiological, therapeutical and experimental evidence supporting this hypothesis. Despite a large body of literature linking aging, inflammation and cancer, convincing evidence for the clear implication of specific inflammatory pathways explaining cancer incidence or mortality during aging is still lacking.

**Abstract:**

Aging is associated with chronic low-grade inflammation, cancer incidence and mortality. As inflammation contributes to cancer initiation and progression, one could hypothesize that age-associated chronic low-grade inflammation contributes to the increase in cancer incidence and/or mortality observed during aging. Here, we review the evidence supporting this hypothesis: (1) epidemiological associations between biomarkers of systemic inflammation and cancer incidence and mortality in older people, (2) therapeutic clues suggesting that targeting inflammation could reduce cancer incidence and mortality and (3) experimental evidence from animal models highlighting inflammation as a link between various mechanisms of aging and cancer initiation and progression. Despite a large body of literature linking aging, inflammation and cancer, convincing evidence for the clear implication of specific inflammatory pathways explaining cancer incidence or mortality during aging is still lacking. Further dedicated research is needed to fill these gaps in evidence and pave the way for the development of applications in clinical care.

## 1. Introduction

### 1.1. Cancer Incidence and Mortality Increase with Age

Chronological aging is associated with higher cancer incidence and cancer-related burden of disability and mortality. Both in Europe and in the USA, all-cancer annual incidence rises from <400 to >2000 per 100,000 persons aged <50 and >70, respectively (European Cancer Information System, Ispra, Italy and SEER Explorer, National Cancer Institute, NIH, Bethesda, Rockville, MD, USA [1]). Worldwide, the current number of cancer-related disability-associated life years rises from <25 per 1000 persons aged <50, to 100 in women and 190 in men aged >70 (Global Health estimates 2019, WHO [2]), and annual cancer-related mortality rises from <0.5 per 1000 persons aged <50 to 7 women and 13 men per 1000 persons aged >70. Cancer is thus considered as an age-related disease.

Data by tumor sites in the USA indicate that, apart from testis, cervix uteri, Hodgkin lymphoma and thyroid, the incidence of most cancers increases quasi-linearly with age from age 40–50 (SEER Explorer, National Cancer Institute, NIH, Bethesda, Rockville, MD, USA [1]). The same tendency is observed for site-specific cancer mortality from age 50–60. Leading causes of both death and disability worldwide after 70 years of age are: respiratory, prostate, colorectal and stomach cancers in men and respiratory, colorectal, breast and stomach cancers in women (Global Health estimates 2019, WHO [2]).

Since age is a strong risk factor common to cancer and several other chronic diseases [3,4], a better understanding of the mechanisms of biological aging could help in identifying the causes of these diseases, including age-associated cancers, and could lead to better care for patients [5]. Inflammation is thought to be a key feature of biological aging that may explain the onset of cancer [6,7].

### 1.2. Aging Is Associated with Chronic Low-Grade Inflammation

Inflammation can be defined as an adaptive response triggered by noxious stimuli and conditions. Inflammatory pathways include sensors such as TLR and NLR, mediators such as interleukine (IL)-1β, IL-6 and tumor necrosis factor (TNF), and effectors such as endothelial cells and leukocytes [8]. The best characterized type of inflammation is an acute high-grade but not age-specific response triggered by infection or tissue injury. On the other hand, a much less understood type of inflammation is chronic, low-grade and age-related [8,9]. The mechanisms, biomarkers and physiological roles of the latter form of inflammation remain poorly understood. The term ‘para-inflammation’ has been proposed to describe an adaptive response to tissue stress or malfunction as an intermediate state between homeostasis (i.e., basal state) and inflammation (i.e., response to infection or injury) [8]. Such inflammation is associated with and thought to contribute to the pathophysiology of most chronic diseases [8,9]. It has also emerged as a hallmark of biological aging [5,10]. While this age-related inflammation and its supposedly negative consequences on health are frequently referred to as ‘inflammaging’ [11], its origins, mechanisms and impact remain largely elusive.

An increase in serum inflammatory cytokines and in vitro production of these cytokines by peripheral blood mononuclear cells or monocytes with age has been reported [12,13], but a closer look at different aging phenotypes reveals a more complex scenario. Studies comparing inflammatory cytokine levels in young and healthy older individuals found conflicting results [14,15,16] and the relationship between age and serum inflammatory cytokines is attenuated when taking chronic diseases into account [13]. Thus, a direct association between physiological aging and systemic inflammation is difficult to establish. More consistently, the serum level of several inflammatory cytokines predicts mortality in older persons [17,18] and is associated with clinical markers of unsuccessful aging. The latter term refers not only to age-related diseases studied separately or pooled as multimorbidity (reviewed in [9,19]), but also to frailty, i.e., the loss of physiological reserve increasing vulnerability to stressors [20] and the loss of independence in activities of daily living [21,22,23]. Complementary to these human epidemiological data, murine models suggest the involvement of specific inflammatory pathways in multi-system functional decline during aging [24,25].

Of note, this age-related chronic low-grade inflammatory profile—and the chronic diseases it is associated with—is specifically associated with the ‘western’ aging trajectory and is not shared by populations with a very different lifestyle as exemplified by several studies of hunter–gatherers (reviewed in [9]). Thus, chronic low-grade inflammation does not seem to be an intrinsic characteristic of biological aging and is probably strongly influenced by environment and lifestyle, especially pollutants, diet and physical activity.

In summary, chronic low-grade inflammation seems to be associated with ‘unsuccessful’ aging (i.e., with age-related diseases, functional loss and mortality), specifically in Western lifestyle contexts, more than with aging itself.

### 1.3. Inflammation Contributes to Cancer Incidence

Several age-related chronic diseases are associated with chronic low-grade inflammation. In particular, inflammation has been recognized as a hallmark of cancer [26], due to its association with aspects of tumorigenesis, like epithelia-mesenchymal transition, proliferation, survival, angiogenesis, invasion and metastasis. Experimental data suggest that inflammation contributes to several stages of carcinogenesis.

The initiation stage is probably the most difficult to study, but evidence from animal models suggests that inflammation promotes genomic instability/DNA mutations (review in [27,28]), particularly through reactive oxygen species produced by activated inflammatory cells [29] and inflammatory cytokines. Of note, DNA damage will in turn exacerbate inflammation and promote tumorigenesis (review in [30]).

At the promotion stage, inflammatory cytokines (notably TNF, IL-1β and IL-6) induce NF-κB and STAT3 transcription factors in cancer cells, which activate genes controlling cell survival, proliferation and growth. Inflammatory cytokines are produced by cancer cells themselves or by immune cells in a paracrine manner (review in [28,31]). Angiogenesis also depends on a paracrine amplification loop between cancer cells and tumor-associated macrophages (TAM).

At the dissemination stage, inflammatory cytokines such as TNF, IL-1β and IL-6 increase the invasive capacity of malignant cells through the upregulation of chemokine receptor expression (review in [30]) and contribute to metastasis by promoting the survival of circulating cancer cells [32]. TAM also contribute to intravasation and the metastatic spread of cancer cells in several models (review in [30]).

### 1.4. Does Inflammation Contribute to Cancer Incidence and Mortality during Aging?

**Figure 1:**
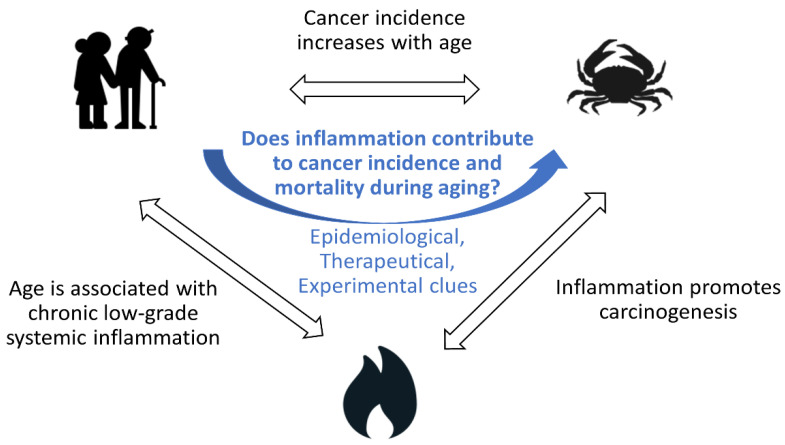
Does age-associated chronic low-grade inflammation contribute to the increase in cancer incidence and/or mortality observed during aging?

Since (1) cancer incidence and mortality increase with age, (2) aging is associated with chronic low-grade inflammation and (3) inflammation contributes to several stages of tumorigenesis, it can easily be hypothesized that age-associated chronic low-grade inflammation contributes to the increase in cancer incidence and/or mortality observed during aging [33,34]; but is this true? In this conceptual review, we summarize the evidence supporting this hypothesis: (1) epidemiological associations between biomarkers of systemic inflammation and cancer incidence and mortality in older people, (2) therapeutic clues suggesting that targeting inflammation could reduce cancer incidence and mortality and (3) experimental evidence from animal models highlighting inflammation as a mechanistic link between hallmarks of biological aging (notably cell senescence, telomere shortening, stem cell exhaustion) and cancer initiation and progression (Figure 1). Then, we discuss the limitations of such data, how research could fill knowledge gaps and how such advances could be translated into clinical care.

## 2. Epidemiological Associations between Systemic Inflammation and Cancer Incidence in Older People

### 2.1. Inflammatory Biomarkers and Cancer Incidence: Summary of Meta-Analyses

Several cohort studies and nested case-control studies have found positive associations between serum markers of inflammation and the incidence of various cancers. The results are summarized in Table 1. Two meta-analyses of such studies found associations between serum C-reactive protein (CRP) and incidence of breast cancer [35,36]. Two other analyses found borderline associations between CRP and IL-6 and colon cancer incidence, but no, or an inverse, association with rectal cancer [37,38]. A meta-analysis of almost 200,000 persons (11,000 cases) found an association between CRP and the incidence of all cancers (and with lung, but not breast, colorectal or prostate cancer taken individually) [39]. Effect size was generally small and most authors found evidence for heterogeneity among studies and/or small study or publication bias.

The median age in these studies was 45 to 73 years of age. Interestingly, in sub-analyses according to age groups three meta-analyses found significant results only in participants older than 60 [36,38,39]. The role of systemic inflammation in cancer incidence might thus be different according to age. These results could also be explained by a higher number of incident cancers in older persons and by a stronger association between inflammatory biomarkers and cancer incidence, both reducing the statistical power needed to observe an association in this age group. Few studies focused on older persons, except a cohort study including 2400 American septuagenarians that found associations between higher CRP, IL-6 and TNF with all cancer incidence [43]. Interestingly, Agnoli et al., reported associations of different serum cytokines with pre- and post-menopausal breast cancers (respectively TNF and CRP), suggesting that the inflammatory pathways linked to cancer incidence depend on age for the same tumor site [47]. In a randomized controlled trial of an anti-IL-1β monoclonal antibody (CANTOS study), originally designed as a secondary prevention trial to reduce cardiovascular events, serum CRP and IL-6 were found to be associated with lung cancer incidence [48] and with time to cancer diagnosis [49].

### 2.2. Inflammatory Biomarkers and Cancer Mortality: Summary of Single Studies

Apart from cancer incidence, several cohort studies also found positive associations between serum inflammatory biomarkers and cancer-related mortality (see Table 1). Biomarkers included CRP, IL-6, leukocytes, neutrophils, haptoglobin, albumin and TNF. Tumor sites were breast, colorectal, lung, prostate or all cancers. The cohorts were built in USA or in Europe and the mean age was around 50–60 years at inclusion, except for one study including only septuagenarians [43]. Overall, associations were stronger with cancer mortality than associations with cancer incidence as described above.

### 2.3. Limitations of These Studies

Besides their modest effect size and bias as in the meta-analyses described above, these epidemiological studies have limitations. Firstly, associations do not mean causality, therefore inflammation might not be mechanistically involved in cancer initiation or progression despite these data. Thus, serum mediators of inflammation can be viewed as risk markers to some extent. Secondly, most of these biomarkers reported in cohort studies are routine clinical practice biomarkers that do not provide clues on specific inflammatory pathways that could contribute to cancer. Thirdly, serum mediators of inflammation do not necessarily provide information about inflammation at the (pre) tumoral site. Finally, regarding associations between inflammatory biomarkers and cancer mortality, death, as an event, encompasses cancer incidence and failure of cancer treatment. Thus, these associations could have several explanations. In addition, the above-mentioned studies used data obtained from cohorts of individuals from the general population. To explore links between systemic inflammation and cancer mortality, a complementary approach is to assess the prognostic value of inflammation biomarkers among cancer patients.

### 2.4. Inflammatory Markers Are Associated with Clinical Outcomes among Cancer Patients

In these settings, inflammation may originate from the tumor itself or the host. Thus, data cited in this section provide clues about how systemic inflammation contributes to cancer prognosis, but not specifically about how this inflammation contributes to cancer progression. Nevertheless, we discuss these data in the present review because they appear more directly translatable to clinical care.

The most studied biomarkers are CRP and albumin, combined in a score referred to as the Glasgow prognostic score. As reviewed by McMillan [50], this score has been used in more than 60 studies (*n* = 30,000 patients) in 13 different countries and in various tumor sites and treatment types. It was reproducibly found predictive of cancer mortality, independently of disease severity. Associations with other clinical parameters and outcomes provide clues about putative mechanisms linking systemic inflammation and mortality in cancer patients. Indeed, the Glasgow prognostic score has been associated with weight and muscle loss, malnutrition, comorbidity and treatment toxicity, all of which might contribute to prognosis. Then, systemic inflammation may be considered as a marker of vulnerability in cancer patients in several ways: as a marker of reduced physiological reserve (e.g., through malnutrition and muscle loss), tumor aggressivity and/or treatment efficacy. To explore the malnutrition hypothesis and to improve our understanding of ‘cancer cachexia’ mechanisms, a recent pooled analysis of three cohort studies assessed relations between CRP levels, food intake, cancer-associated weight loss and mortality in 12,253 patients (mean age: 65 y). Higher CRP was associated with weight loss independently of food intake; both higher CRP and reduced food intake independently predicted mortality [51]. This suggests that systemic inflammation might contribute to weight loss and mortality, independently of its known anorexia effect.

Given the growing interest in oncogeriatric research, more recent studies specifically focused on the prognosis of inflammatory biomarkers in older patients with cancer. In this special issue of *Cancers*, Brattinga et al., report a significantly higher mortality rate three years after surgery in patients (median age 71.5) with pre-operative higher CRP or IL-6 plasma levels. This association was independent of age, disease stage, comorbidities, type of surgery and complications [52].

## 3. Therapeutic Approaches Targeting Inflammation to Reduce Cancer Incidence

Evidence that drugs with an anti-inflammatory effect are associated with a reduction in cancer incidence or mortality are summarized in Table 2. A meta-analysis of observational studies including 19 case control studies and 11 cohorts yielded an association between regular use of aspirin or non-steroidal anti-inflammatory drugs (NSAID) and a lower first colorectal cancer incidence 10 years after exposure. Associations were consistent only when the daily dose was over 300 mg [53]. Aspirin is thought to affect the development of tumors by inhibiting COX-2 [54], which is highly expressed in colorectal neoplasia [55]. A more recent cohort study of postmenopausal women confirmed the association between NSAID use and a reduced incidence of colorectal cancer, but no reduction in total cancer incidence. The investigators also reported reductions in ovarian cancer and melanoma incidence, although this needs confirmation [56]. We thus have clues that the long-term use of anti-inflammatory drugs may reduce cancer incidence, especially in the case of colorectal cancer. Nevertheless, the exact mechanisms of these putative effects remain unknown. Observational studies offer a low level of evidence notably due to information bias on drug use and the benefit/risk ratio of long-term exposure to high-dose aspirin or NSAIDs is not clear.

Stronger evidence is provided by secondary analyses of randomized controlled trials (RCT) of anti-inflammatory drugs. Most data are from studies of the cardiovascular preventive effect of aspirin. A meta-analysis of two British RCT of aspirin (five years of use, 300 to 1200 mg vs. placebo) revealed a reduction in first colorectal cancer incidence only after 10 years of use [53]. Another meta-analysis of four European RCT (the two previously cited RCT and two additional RCT comparing 75 mg daily aspirin vs. placebo) indicated a reduction in the 20-year incidence of colon (but not rectal) cancer incidence and mortality. This effect was observed with a treatment duration greater than five years. The benefit did not increase with doses higher than 75 mg/d. A 2% absolute reduction in 20-year fatal cancer risk was calculated [57].

Besides these data focusing on colorectal cancer, two meta-analyses of 8 and 51 RCT, respectively, showed a reduction in total cancer risk in participants taking aspirin [58,59]. Of particular interest for the hypothesis of the present review, one of these studies highlighted a greater reduction in 20-year cancer mortality in older patients. The reduction in absolute 20-year risk of death due to any cancer in the aspirin groups were 1.41% at age less than 55 years, 4.53% at age 55–64 years and 7.08% at age 65 years or older, especially due to differences between age groups for non-gastrointestinal cancers. The effect on all-cancer mortality was due to effects on lung, colorectal and esophageal cancer, and no effect was observed on other cancer mortality [59]. We thus have therapeutic clues that long-term use of aspirin could reduce cancer incidence and mortality, especially for colorectal cancer and with a greater effect in older persons.

Nevertheless, low doses of aspirin (75–100 mg) are not usually considered as anti-inflammatory and the mechanisms of their putative effect on cancer risk reduction are still elusive. Non-inflammatory mechanisms, both COX-dependent and independent, have been hypothesized to explain the anti-cancer properties of aspirin, including effects on apoptosis, DNA-mismatch repair, migration and angiogenesis, possibly involving platelets and microbiota [61,62].

Recent data suggest that the specific inhibition of IL-1β with a monoclonal antibody could reduce cancer risks. CANTOS was a secondary cardiovascular prevention trial in patients with persistent low-grade inflammation after myocardial infarction [63]. In addition to reduced CRP and IL-6 levels, cardiovascular events and death, canakinumab treatment lowered lung cancer incidence and mortality as well as total cancer mortality vs. placebo. Nevertheless, fatal infections were increased in the treated groups and all-cause mortality was not decreased, highlighting the fact that inhibiting inflammatory pathways does not have only beneficial effects. Further research is needed to optimize inhibition of the inflammatory pathways involved specifically in age-related diseases.

Observational studies and RCT thus suggest that inhibiting inflammation may reduce cancer incidence and mortality, especially in colorectal and lung cancer. Despite these promising data, none of these studies were initially designed to study cancer and few of them concentrated on older patients; the underlying mechanisms of these effects are still poorly understood and these data have not yet been translated into clinical care.

## 4. Experimental Evidence Linking Hallmarks of Biological Aging and Cancer through Inflammation

Animal models may provide more insight into the mechanisms linking inflammation to tumorigenesis during aging. Biological aging is a highly complex and multifaceted process that remains difficult to study comprehensively. Thus, experimental research is frequently focused on one of its specific aspects, recognized as the hallmarks of aging [5,10]. In this section, we summarize experimental evidence that inflammation may constitute a mechanistic link between specific aspects of biological aging and tumorigenesis. With this aim in mind, we searched the literature for models in which experimental manipulation of hallmarks of biological aging influenced cancer initiation or progression in an inflammation-dependent manner.

### 4.1. DNA Damage

The accumulation of DNA damage is frequently suggested as a major cause of increasing cancer incidence with aging. DNA replication errors are thought to be responsible for two-thirds of the mutations in human cancers (data from 17 cancers in 69 countries, [64]. Inflammation can be seen both as a cause and a consequence of DNA damage (see [65] for review). In vitro, Rodier et al. [66] showed that persistent chromatin lesions bearing hallmarks of DNA double-strand breaks initiate increased secretion of inflammatory cytokines such as IL-6 and that the DNA damage-response protein ATM is essential for the ability of senescent cells to stimulate IL-6-dependent cancer cell invasiveness. However, to our knowledge, in vivo models suggesting that inflammation is involved in DNA damage-induced carcinogenesis are lacking.

### 4.2. Telomere Shortening

Telomere shortening is a specific type of genomic damage observed during normal aging in mice and humans [67]. Telomeres are repetitive DNA sequences capping chromosomes, which become shorter at each cell division. Experimental modification of telomere loss influences the lifespan of mice [68,69]. By inducing replicative senescence, telomere shortening may be viewed as a tumor suppressor mechanism; nevertheless, mice with accelerated telomere shortening exhibit more tumors [70,71]. To better understand the role of telomere shortening in the tumor environment i.e., the host, Lex et al. used a zebrafish chimeric model in which the host, but not the tumor, lacked telomerase reverse transcriptase, leading to accelerated telomere shortening. Markers of senescence and inflammation (expression of *p15/16* and *Tnf*, respectively) and invasion/progression of melanoma tumors were increased in these hosts compared to wildtype fishes. This phenotype was reversed by using anti-inflammatory drugs [72]. This suggests that inflammation could play a mechanistic non-cell autonomous role linking telomere shortening to cancer progression.

### 4.3. Cell Senescence

Cell senescence is another hallmark of biological aging thought to be a cause of age-related chronic low-grade inflammation. It is described as a stable arrest of the cell cycle coupled to the production of several molecules (including inflammatory cytokines) known as the senescence-associated secretory phenotype (SASP), which facilitates tumor cell growth [73]. Again, even if cell senescence may be viewed as a tumor suppressive mechanism, the accumulation of senescent cells has both deleterious and beneficial effects on tissues and organisms, including tumorigenesis [34]. Of note, the selective elimination of senescent cells attenuates the deterioration of several organs during aging and extends lifespan in mice [74] and the potential anti-aging effect of senolytic drugs is a promising area of research [75].

Using a murine model of stromal-specific inducible senescence, Ruhland et al., demonstrated that stromal senescence increases the expression of pro-inflammatory cytokines and chemokine genes (notably IL-6) and the presence of myeloid-derived suppressor cells. These cells reduce the proliferation and effector functions of CD8+ T cells and facilitate tumor growth following skin injection of cancer cells in an IL6-dependent manner. Interestingly, the authors also reported the accumulation of senescent cells, IL-6 and myeloid-derived suppressor cells during human skin aging [76]. Thus, inflammation may be a mechanistic link between cell senescence and a reduction in immunosurveillance contributing to tumor progression.

Links between senescence, inflammation and tumorigenesis were further highlighted by Pribluda et al., in a murine model of colorectal cancer. In this work, enterocyte-specific *CKIα* deficiency triggered a ‘senescence-associated inflammatory response’, leading to growth arrest when the tumor suppressor gene *p53* was functional, but to carcinogenesis in the absence of *p53* (double knockout, KO). Experiments in pure epithelial organoid cultures derived from these mice suggested that this phenomenon is cell autonomous without the involvement of microbiota or immune cells. Furthermore, anti-inflammatory drugs decreased tumor burden in double KO mice and reversed proliferation of double KO organoids. This illustrates the principle that carcinogenesis can be induced by low-grade inflammation resulting from tissue stress, in line with the concept of ‘para-inflammation’ [77] (and see comment in [78]). It also suggests that inducing a senescence-associated inflammatory response is not sufficient to trigger tumorigenesis in every model.

### 4.4. Stem Cell Dysfunction

Stem cell dysfunction is recognized as a hallmark of aging and the aging features of hematopoietic stem cells (HSC) include inflammation and a propensity to myeloid malignancy. Grants et al. [79] reported that microRNA-146a (miR-146a) levels were inversely correlated with the activation of IL-1, IL-6 and TNF pathways in 102 adult patients with acute myeloid leukemia. Low miR146a expression was predictive of mortality in this cohort. The authors also found a decline in HSC miR146a expression during aging in wildtype mice and described miR146a−/− mice that exhibited HSC characteristics close to HSC aging, including expression of *Tnf* and *Il6* via the NF-κB and JAK STAT3 pathways, respectively. Survival of these mice was reduced due to hematological malignancy in an *Il6*- and *Tnf*-dependent manner. In addition to a bone marrow inflammatory milieu in miR146a−/− mice, cell-intrinsic signals probably contributed to carcinogenesis, since miR146a−/− HSC were more sensitive to IL-6-mediated rupture of quiescence. This suggests that IL-6 and TNF pathways may contribute to myeloid malignancy during age-associated HSC dysfunction.

In the same line, Henry et al. [80] described signaling, gene expression and metabolic defects in B cell progenitors from old mice on the one hand, and young HSC transferred to old mice on the other. Aging was associated with increased inflammation in the bone marrow microenvironment and the induction of inflammation in young mice phenocopied aging-associated B cell progenitor defects. A genetically driven reduction in inflammation in aged mice preserved the function of B cell progenitors and prevented oncogenesis. Thus, bone marrow inflammation may contribute to B cell progenitor dysfunction, leading to the selection of cells with oncogenic mutations. Of note, inflammatory cytokine (i.e., IL-6, TNF) levels found in serum were not totally similar to bone marrow levels, especially when considering the effects of the genetically driven reduction in inflammation. This suggests that systemic inflammation may not always reflect processes involved in tissues in which cancers develop.

## 5. Conclusions

In this review, we have summarized the epidemiological, therapeutical and experimental evidence that inflammation might contribute to cancer incidence and mortality during aging. Serum levels of inflammatory markers like CRP, IL-6 and TNF are associated with the incidence of several cancers and mortality in cohorts and meta-analyses in the general population, with a modest effect size. Inflammatory markers also have a prognostic value among cancer patients in various settings. Long-term aspirin use is associated with lower cancer incidence and mortality, especially for colorectal cancer and in older people, but the mechanism of this putative effect remains elusive. Lung cancer incidence and mortality might be reduced by IL-1β inhibition, but this is counterbalanced by the incidence of fatal infections and needs confirmation in other studies. Animal models provide clues into how inflammation might link hallmarks of biological aging (especially telomere shortening, cell senescence and stem cell dysfunction) to cancer initiation and progression; however, they are not easily translated into understanding the exact role of inflammation in age-associated cancers as hallmarks of aging taken separately do not reproduce the complexity of the human aging process. Altogether, these data support the putative effect of aging-associated inflammation on the development of cancer in old age, but they fail to provide final evidence; they provide neither clear evidence of causality nor clues into the specific inflammatory pathways involved in age-related cancers. Thus, it is too early to state that inflammation contributes to age-related cancers, although it is obvious for physicians that tumors themselves produce inflammation that can be detected in clinical practice.

This lack of evidence for the role of inflammation in age-related cancers may be in part related to the vagueness of the concept of age-related inflammation and how it is measured. While inflammation in older persons is usually considered as chronic and low grade, most studies cited in this review relied on single measures of inflammatory markers, therefore the term ‘chronic’ may not be appropriate; furthermore, the term ‘low grade’ has never been precisely defined. Secondly, is age-related inflammation supposed to be local or systemic? Epidemiological studies present only systemic inflammatory markers, while animal model studies have mostly concentrated on local inflammation. The study by Henry et al. [80] on B cell progenitors is an exception in that it reported both bone marrow and serum inflammatory cytokines and suggested that systemic inflammation may not always reflect processes involved in tissues in which cancers develop. As suggested by a recent comment [81] ‘tissues, not blood, are where immune cells act’ and should be studied. Thirdly, evidence is lacking that the putative role of inflammation in cancer initiation or progression is specific to older age. Sub-analyses of the few studies cited above showed that associations between inflammatory biomarkers and cancer incidence might be specific to older patients [36,38,39] and that long-term use of aspirin could reduce cancer incidence and mortality with a greater effect in older persons [59]. Nevertheless, only systematic comparisons of different age groups in such studies, as well as animal models investigating the involvement of inflammatory pathways to cancer at different ages, would help in determining whether the contribution of inflammation to cancers is age specific.

**Figure 2:**
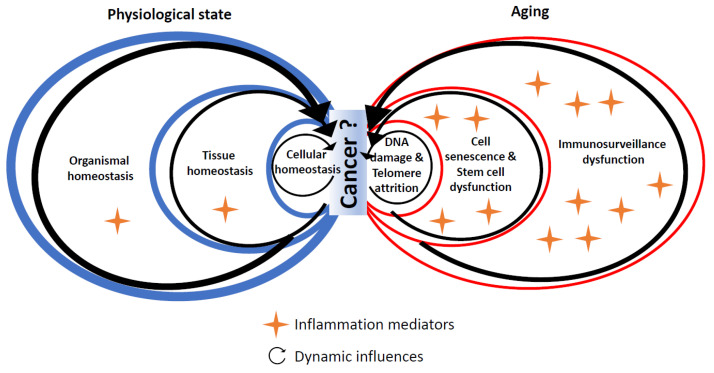
Interplay of factors associated with homeostasis, aging and inflammation in cancer development.

Given these limitations and the lack of strong evidence, we propose the following perspective (Figure 2), derived from the concept of para-inflammation [8]: the physiological state relies on homeostasis at the cellular, tissue and organismal levels. Age-related cancers are characterized by a loss of homeostasis and inflammation at these three levels: DNA damage and telomere attrition at the cellular level, cell senescence and stem cell dysfunction at the tissue level and dysfunctional immunosurveillance at the tissue and organismal levels. At which level and how this shift occurs remain poorly known.

One could hypothesize that the oxidative stress (especially ROS production) and mitochondrial dysfunction observed during aging could be a source of age-related inflammation and could contribute to cancer; indeed, inflammation increases ROS production and ROS are drivers of carcinogenesis, notably through DNA damage [29,82]. Furthermore, mitochondrial dysfunction is involved in inflammasome activation following various stimuli [83]. Nevertheless, we did not include oxidative stress as a putative hallmark of aging linked to cancer through inflammation in the present review since: (a) we are not aware of any experimental data suggesting inflammation as a mechanistic link between oxidative stress and cancer, (b) a reduction in mitochondrial ROS was observed in aged B cells in the above-cited model of stem cell dysfunction leading to oncogenesis though inflammation [80].

Importantly, inflammation is not the only aspect of the immune system that may link aging to cancer. The role of immunocompetent cells in controlling the development of tumors is now well established; a process referred to as ‘cancer immunosurveillance’ [84]. By virtue of their effector functions and ability to recognize cancer cells, CD8+ T cells, γδ T cells and NK cells can eliminate malignant cells. Moreover, proinflammatory monocytes and macrophages and T regulatory cells and myeloid-derived suppressor cells can directly modulate these effector responses. However, aging is associated with both quantitative and qualitative alterations of these immunocompetent cells [85,86]. Furthermore, age-related changes of their environment (e.g., increased levels of senescent cells, levels of systemic inflammation, activation of inflammasomes) may directly affect the functional properties of these cells. Such alterations of immunocompetence with increasing age, which is often referred to as ‘immunosenescence’, may therefore result in ineffective anti-tumor immunosurveillance. For instance, old individuals were less able to induce a de novo CD8+ T cell response against a tumor antigen in an in vitro experimental study [87]. Although hypothetical at this stage, defective immunosurveillance rather than inflammation directly may thus contribute significantly to cancer incidence and mortality during aging [88]. Some models have attempted to integrate the effects of age-related changes of inflammation and immune cell functions on cancer development. In the above-cited work by Ruhland et al. [76] cell senescence and tumor progression are linked through IL-6 secretion, MDSC recruitment and dampened CD8+ T cell proliferation and effector functions. On the other hand, other models suggest that SASP components may stimulate cancer immunosurveillance. For example, Ianello et al. found that p53-dependent chemokine production by senescent tumor cells supports NKG2D-dependent tumor elimination by natural killer cells [89]. Thus, depending on the model, biological age-related inflammation may have an opposite effect on cancer progression through pro- or anti-tumor effects. To date, the contribution of age-related altered immunity to the increased prevalence of solid or liquid cancers in the elderly is an unanswered question of key importance. In line with this, we currently lack markers of immunosenescence that could be used in clinical practice, especially to help decision making in immunotherapy for cancer in older patients. Indeed, knowledge of immunosurveillance mechanisms has been translated into cancer care with the advent of immune checkpoint inhibitors, i.e., drugs that improve progression-free survival in several cancer types [90]. These drugs are currently as efficient, or less efficient, in older than in younger patients depending on the tumor site [91], but we lack biomarkers predictive of patients’ response to these drugs. More generally, as highlighted in a recent review, differences in cancer biology according to age seem clearer regarding histology and mutational landscape than immunological features [92].

How could we better understand the role of inflammation in age-related cancers? Experimental data come from animal models of an accelerated hallmark of aging (e.g., the lack of telomerase reverse transcriptase for telomere shortening or inducible cell senescence). They provide insights into the molecular and cellular consequences of the biological changes that occur during aging, but do not recapitulate the biological complexity of ‘natural’ aging. As demonstrated by Golomb and al., [93], it is feasible to compare the effect of an oncogene mutation on cancer incidence in young vs. old animals and to use the findings to study several biological hallmarks of age-related cancers such as senescence, inflammation and immunosurveillance. However, studying the effect of experimental modifications of inflammatory pathways on cancer initiation and/or progression during natural aging would provide more evidence about the contribution of age-related inflammation to cancers. Such studies should also compare local and systemic inflammatory markers to better characterize this inflammation and pave the way for useful biomarkers. More longitudinal human studies focused on older persons (with and without cancer, with and without chronic low-grade inflammation, in youngest-old, middle-old and oldest-old subgroups) would be helpful to better understand the links between inflammation and prognosis and ultimately to improve clinical care in this specific population.

## Figures and Tables

**Table 1:** Associations of blood inflammatory markers with cancer outcomes.

Cancer	Biomarker	N Participants/*n* Cases	Countries	Design	Mean of Median Age at Inclusion	Effect Size	Heterogeneity and Bias (for Meta-Analysis)	Follow-Up	Reference
**Meta-analyses focused on incidence**									
Breast	CRP	69,610/3522	America (*n* = 6)/Europe (*n* = 5)/Asia (*n* = 1)	12 studies	49–73 y 9 studies restricted to post-menopausal women	RR per doubling CRP concentration: 1.07 (95% CI: 1.02–1.12) 1.06 (1.01–1.11) for post-menopausal women	I² = 47%, Ph = 0.04 Egger’s tests showed some evidence of publication or small study bias (*p* = 0.08)	5–13 y	[35]
Breast	CRP	/5286	America (*n* = 6) Europe (*n* = 6)/Asia (*n* = 3)	15 cohorts + case-control studies	45–73 y	Combined OR per natural log unit change in CRP: 1.16 (95% CI: 1.06–1.27) Only significant in persons >60 y (6 studies)	I2 = 45.9% No evidence of publication bias, Begg’s (0.805) and Egger’s tests (0.172)		[36]
Colorectal	IL6	8420/1308	USA (*n* = 5), UK (*n* = 2)	3 cohorts and 3 nested case-control studies		summary RR per natural log unit change in IL6: 1.22 (95 % CI 1.00–1.49) Inverse association was noted for rectal cancer (RR 0.69; 95 % CI 0.54–0.88)	Some evidence of heterogeneity (I^2^ = 46 %). No evidence of small study effect, Egger’s *p* 0.77 I^2^ = 0 %. Evidence for small-study effects (*p* 0.02).		[37]
Colorectal	CRP	1159 cases/37,986 controls	USA (*n* = 3), Europe (*n* = 3), Japan (*n* = 2)	3 cohort and 5 nested case-control studies	53–73 y	Summary RR per one unit change in natural log-transformed high-sensitivity CRP: 1.13 (95% CI, 1.00–1.27) for colon cancer, and 1.06 (95% CI, 0.86–1.30) for rectal cancer Only significant in persons >60 y (3 studies)	P_h_ 0.16 (colon) and 0.02 (rectal)	5.5–14 y	[38]
All	CRP	194,796/11,459	USA (*n* = 5), Europe (*n* = 6)	11 cohort studies	45–73 y	Pooled HR per natural log unit change in CRP: 1.105 (95% (CI): 1.033–1.178) for all cancers 1.308 (95% CI: 1.097–1.519) for lung cancer Not significant for breast, prostate and colorectal separately Only significant in persons >60 y (*n* = 24,000)	Substantial heterogeneity across studies (P_h_ = 0.000, I2 = 70.10%)		[39]
**Single studies focused on cancer mortality**									
Colorectal	haptoglobin	325,599/1467	Sweden	Cohort	Mean (SD) 46 (14)	Adjusted HR (>1.2 vs. <0.9 g/L): 1.19; 95% CI: 1.01–1.41	No significant association with CRP and albumin	18 y (mean)	[40]
Breast	haptoglobin	155,179/736	Sweden	Cohort	Mean (SD) 50 (11)	Adjusted HR (>1.4 vs. <1.4 g/L): 1.27, 95% CI: 1.02–1.59	No significant association with CRP and albumin	18 y (mean)	[41]
Breast, lung, all	leukocytes	143,748/3062	USA	Cohort	63 (50–79)	Adjusted HR: (higher vs. lower quartile) 2.16 (1.33–3.50) for breast 1.65 (1.29–2.12) for lung 1.33 (1.17–1.51) for all	Not significant for endometrial and colorectal	8 y (mean)	[42]
All	CRP, IL6, TNF	2438	USA	Cohort	73 (70–79)	Adjusted HR (1-unit increase on natural log scale): 1.63 (1.19–2.23) for IL-6 1.64 (1.20–2.24) for CRP 1.82 (1.14–2.92) for TNF		5.5 y (mean)	[43]
All	CRP, albumin, neutrophils	160,481/13,173	UK	Cohort	35% > 65 y	Adjusted HR 1.85 for CRP > vs. <10 mg/L (*p* < 0.001) 2.08 for albumin < vs. >35 g/L (*p* < 0.001) Neutrophils > vs. < 7.5 × 10^9^/L (*p* < 0.001)		69 months (mean)	[44]
Prostate	Leukocytes, neutrophils	210,000/323	UK	Cohort	Mean (SD) 57 (8)	Per one SD increase: HR = 1.14, 1.05–1.24 for leukocytes HR = 1.27, 1.09–1.48 for neutrophils		6.8 y	[45]
Colorectal	CRP	16,000/92	USA	Cohort	50	Adjusted HR = 3.96 (95% CI, 1.64–9.52) for levels >1.00 vs. <0.22 mg/dL		14.2 y	[46]

CI—confidence interval. CRP—C reactive protein. HR—hazard ratio. IL6—interleukin-6. TNF—Tumor necrosis factor. OR—odds ratio. RR—relative risk. SD—standard deviation. UK—United Kingdom. USA—United States of America.

**Table 2:** Studies of effect of anti-inflammatory drugs on cancer incidence and mortality.

Cancer	Drug	Outcomes	N	Country	Design	Age at Inclusion	Reference
**Randomized controlled trials**							
Lung/ All	Canakinumab	Reduction in - hsCRP and IL6 levels - total cancer mortality - lung cancer mortality - incident lung cancer - but not all cause mortality (fatal infections were increased)	10,061		Secondary analyses of a RCT	Mean (sd) 61 (10)	[48]
Colorectal	Aspirin	Cancer incidence reduction (only after 10 years) No effect on incidence of other cancers	5000 + 2500	UK	2 RCT	Mean (sd) 62 (7)/ 60 (9)	[53]
Colorectal	Aspirin	20 y incidence and mortality reduction (colon but not rectum) Effect when treatment duration >5 years. No increase in benefit for dose >75 mg/d 2% absolute reduction of 20 y fatal cancer risk	14,000	UK, Sweden	Meta-analysis of 4 RCT		[57]
All	Aspirin	Reduction in - cancer incidence (after 3 y) - cancer death (particularly after 5 y)			51 RCT		[58]
All	Aspirin	Reduction in 20-year cancer death Benefit increased with age and duration of treatment (but unrelated to aspirin dose, starting at 75 mg)	25,000		8 RCT		[59]
**Observational studies**							
Colorectal	Aspirin or NSAID	Associated with lower colorectal cancer incidence Especially after 10 y of use, Aspirin dose: consistent effect only when dose > 300 mg/d No systematic effect of age on associations	21,000 cases + 1,136,110 (6000 cases)		Meta- analysis 19 case-control studies + 11 cohorts		[53]
All (women)	NSAID (0 and 3 years)	Associated with lower colorectal, ovarian cancer and melanoma incidence (but not with total cancer incidence)	1,290,000	USA	Cohort	50–79 yo	[56]
**Observational studies and randomized controlled trials**							
All	Aspirin	Associated with reduced proportion of cancers with distant metastasis but not with any reduction in regional spread	329 + 5984		Meta-analyses of 3 RCT and 5 observational studies		[60]

CRP—C reactive protein. NSAID—non-steroidal anti-inflammatory drugs. RCT—randomized controlled trial.
